# IL‐22‐mediated renal metabolic reprogramming via PFKFB3 to treat kidney injury

**DOI:** 10.1002/ctm2.324

**Published:** 2021-02-23

**Authors:** Wei Chen, Yilan Shen, Jiajun Fan, Xian Zeng, Xuyao Zhang, Jingyun Luan, Yichen Wang, Jinghui Zhang, Si Fang, Xiaobin Mei, Zhen Zhao, Dianwen Ju

**Affiliations:** ^1^ School of Pharmacy and Minhang Hospital Shanghai Engineering Research Center of Immunotherapeutics Fudan University Shanghai P. R. China; ^2^ Department of Ophthalmology Stanford University School of Medicine Palo Alto California USA; ^3^ Changhai Hospital Second Military Medical University Shanghai P. R. China; ^4^ Department of Nephrology Shanghai Jiao Tong University Affiliated Sixth People's Hospital Shanghai P. R. China; ^5^ Tongcheng Hospital of Traditional Chinese Medicine Anhui P. R. China

**Keywords:** interleukin‐22, kidney injury, metabolic reprogramming, mitochondrial dysfunction

## Abstract

Kidney damage initiates the deteriorating metabolic states in tubule cells that lead to the development of end‐stage renal disease (ESTD). Interleukin‐22 (IL‐22) is an effective therapeutic antidote for kidney injury via promoting kidney recovery, but little is known about the underlying molecular mechanisms. Here, we first provide evidence that IL‐22 attenuates kidney injury via metabolic reprogramming of renal tubular epithelial cells (TECs). Specifically, our data suggest that IL‐22 regulates mitochondrial function and glycolysis in damaged TECs. Further observations indicate that IL‐22 alleviates the accumulation of mitochondrial reactive oxygen species (ROS) and dysfunctional mitochondria via the induction of AMPK/AKT signaling and PFBFK3 activities. In mice, amelioration of kidney injury and necrosis and improvement of kidney functions via regulation of these metabolism relevant signaling and mitochondrial fitness of recombinant IL‐22 are certificated in cisplatin‐induced kidney damage and diabetic nephropathy (DN) animal models. Taken together, our findings unravel new mechanistic insights into protective effects of IL‐22 on kidneys and highlight the therapeutic opportunities of IL‐22 and the involved metabolic regulators in various kidney diseases.

## Graphical abstract

IL‐22 attenuates kidney injury via metabolic reprogramming of renal TECs, regulates mitochondrial function and glycolysis in damaged TECs, and alleviates the accumulation of mitochondrial ROS and dysfunctional mitochondria via the induction of AMPK/AKT and PFBFK3 activities. Ameliorating kidney injury of IL‐22 via regulation of metabolism relevant signaling is certificated in cisplatin‐induced kidney damage and diabetic nephropathy animal models.

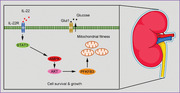

**Abbreviations**
AKI: acute kidney injury
BUN: blood urea nitrogen
Cr: creatinine
DN: diabetic nephropathy
ECAR: extracellular acidification rate
ESTD: end‐stage renal disease
HFD: high‐fat diets
IL‐22: interleukin‐22
MRC: maximal respiratory capacity
OCR: oxygen consumption rate
OXPHOS: oxidative phosphorylation
PKM2: S‐nitrosylation of pyruvate kinase M2
PTECs: proximal tubular epithelial cells
RNA‐seq: RNA sequencing analysis
ROS: reactive oxygen species
TECs: renal tubular epithelial cells

## 1 INTRODUCTION

Acute kidney injury (AKI), a common public health concern associated with high mortality and morbidity, affects millions of hospitalized patients worldwide and shows a fast‐increasing incidence.^1^, ^2^, ^3^ The underlying pathophysiology of AKI is not fully understood but involves the damage and apoptosis of renal tubular epithelial cells (TECs), especially in the proximal tubule.^4^, ^5^, ^6^, ^7^ Multiple literatures support that their severe and sustained damage often causes incomplete and maladaptive tissue repair, leading to inflammatory response, tubular degeneration, kidney fibrosis, and eventual progression to end‐stage renal disease (ESTD).^8^, ^9^, ^10^ Unfortunately, other than dialysis‐based supportive care, no well‐established therapeutics for kidney injury are available. Therefore, proactive treatment is much needed to reduce the suffering it causes to patients and the significant financial burdens of kidney injury to individuals and society.

Interleukin‐22 (IL‐22), an important member of the IL‐10 family cytokines, has recently attracted tremendous attention as a survival agent in numerous disorders driven by epithelial damage.^11^, ^12^, ^13^ IL‐22 elicits tissue protection and homeostasis primarily via activation of the STAT3 signaling pathway and the promotion of epithelial proliferation.^14^, ^15^ Importantly, studies have demonstrated that the expression of IL‐22R1 is limited to renal proximal TECs, and treatment with IL‐22 can protect against renal epithelial injury and accelerate tubular regeneration.^16^, ^17^ However, although accumulating evidence indicates IL‐22 is an effective therapeutic antidote for kidney injury, little is known about the underlying mechanisms of IL‐22‐induced TECs recovery. Understanding the molecular basis of IL‐22 is significant both for exploring how IL‐22 acts to inhibit AKI or ESTD and for discovering molecular modifiers as well as crucial processes involved in preventing kidney damage.

Kidney repair after the damage is a metabolically dependent and complicated process.^18^ Recent evidence indicates that cell metabolic signatures can regulate renal cell survival and plasticity and thus provide useful mechanistic insights into how the metabolic processes control tissue repair.^19^, ^20^ Specifically, these findings show that metabolic reprogramming by PGC1α or the S‐nitroso‐CoA reductase system protects against kidney injury. Mechanistically, increased glycolysis and oxidative phosphorylation (OXPHOS) can promote growth and defensive signaling pathways or can supply the higher energetic demands of mitosis and anabolic biosynthesis during organ repair.^19^, ^21^ Recently, our studies have demonstrated that IL‐22 alleviates mitochondrial dysfunction in the liver, which involves the cellular metabolic processes.^22^, ^24^ Thus, we ask whether IL‐22 protects against kidney damage and apoptosis via regulating their metabolic states. To determine the mechanisms of IL‐22 mediated kidney repair, we treat human proximal tubular epithelial cells (PTECs) with stress stimulation. We suggest that IL‐22 drives a metabolic reprogramming to enhance glycolysis and OXPHOS, which prevent against TECs dysfunction. We further demonstrate that IL‐22 induced this program via activation of AMPK/AKT signaling and its molecular modifiers to alleviate mitochondrial dysfunction. Our observations shed light on regulating metabolic states for treating and preventing kidney diseases, and provide new insights for developing efficient novel interventions for organ injury in general.

## 2 MATERIALS AND METHODS

### 2.1 Reagents

Oligomycin, rotenone, and cyanide p‐trifluoromethoxyphenyl‐hydrazone (FCCP) were obtained from Seahorse Biosciences; recombinant IL‐22 was provided by Novoprotein (China); cisplatin (Cisp.), MitoTracker Green, LY294002, 5,5′,6,6′‐tetrachloro‐1,1′,3,3′‐tetraethylbenzimidazolylcarbocyanine iodide (JC‐1), and compound C were obtained from Beyotime Biotechnology (China); antibodies targeting Glut1, β‐Actin, p‐AMPKα, AMPK, and GAPDH were purchased from Abcam; antibodies for STAT3, p‐STAT3 (Y705), AKT, p‐AKT (S473), PFKFB3, and Capase‐3 were purchased from Cell Signaling Technology; rapamycin, palmitic acid (Palm.), Z‐VAD‐FMK, and glucose were obtained from Sigma‐Aldrich; MitoSOX, Hoechst33342, MitoTracker Red, and PKH26 were obtained from Invitrogen.

### 2.2 Cell culture

Human proximal tubule epithelial cell (HK‐2) was obtained from Cell Bank of Shanghai Institute of Biochemistry and Cell Biology, Chinese Academy of Sciences and maintained following standard protocols. HK‐2 was seeded in six‐well plates (1 × 10^6^ cells/well) or 96‐well plates (1 × 10^5^ cells/well) and cultured in 90% RPMI1640 medium (Gibco) supplemented with 100 U/ml penicillin, 100 µg/ml streptomycin, and 10% fetal bovine serum (FBS) (Gibco). We first incubated HK‐2 with IL‐22 (0.5 µg/ml) for 0.5 h, then 50 mM d‐glucose or 5 µg/ml cisplatin or 0.2 mM palmitic acid or or 4 µM doxorubicin (DOX) for 24 h. In some cases, cells were cultured in the presence of Z‐VAD‐FMK (10 µM), compound C (5 µM), LY294002 (20 µM), or rapamycin (50 nM) for indicated times.

### 2.3 Seahorse experiments

We analyzed TECs using a Seahorse XF96 Extracellular‐Flux‐Analyzer for the changes in the extracellular acid rate (ECAR) and oxygen consumption rate (OCR). Briefly, HK‐2 (1 × 10^5^) was grown on 96‐well Seahorse‐plates for overnight. Then, we washed cells with seahorse assay medium containing 1 mM pyruvate, 10 mM glucose, and 2 mM glutamine (or glucose free for testing ECAR) in an atmosphere without CO_2_ at 37°C for 0.5 h. Experimental data were recorded after the undermentioned inhibitors were injected at an optimum concentration of FCCP (1.0 µM), oligomycin (1.0 µM), rotenone/antimycin A (0.5 µM), 2‐DG (50 mM), or glucose (10 mM).

### 2.4 Mice and animal models

Male BALB/c mice (6–8 weeks) were purchased from Slaccas Experimental Animal Co. (Shanghai, China). Db/db and Db/m mice were provided by Model Animal Research Center of Nanjing University (Nanjing, China). All mice were kept in specific pathogen‐free (SPF) facilities and grown following standard protocols. For the AKI model, BALB/c mice received cisplatin (20 mg/kg) or saline control by intraperitoneal injection. Db/db mice were fed with high‐fat‐diet (HFD) at the indicated time points to induce diabetic nephropathy (DN). Db/m mice were fed with chow‐diets as the control group. In AKI animal experiments, IL‐22 was intraperitoneal injected at the dose of 0.5 mg/kg/day or 1.5 mg/kg/day for 4 consecutive days. In DN animal experiments, IL‐22 was intraperitoneal injected at the dose of 2.5 mg/kg twice weekly for eight consecutive weeks. The control mice received the appropriate amount of PBS. Our animal experimental procedures were carried out following the protocols evaluated and approved by the Animal Care and Use Ethics Committee of School of Pharmacy, Fudan University. Immunohistochemical staining, measurement of mitochondrial membrane potential, histological, and reactive oxygen species (ROS) staining of kidney sections were performed as previously described.^22^, ^23^, ^24^

### 2.5 Real‐time PCR

We obtained the total‐RNA by TRNzol reagents (Beyotime Biotechnology, China) from cell or tissue samples and then transcribed to cDNA using a MMLV‐reverse transcriptase test kit (Beyotime Biotechnology). The expression level of mRNA was analyzed by the real‐time PCR instruments (BioRad) using SYBR green qPCR‐mix assay kit (Beyotime Biotechnology) and standardized to GAPDH.

### 2.6 Flow cytometry

Kidney cells were grown and treated as described above. MitoSOX (mitochondrial ROS), MitoTracker Green (total mitochondrial mass), and MitoTracker Red (mitochondrial membrane potential) staining were carried out according to the manufacturer's instructions and previous research.^22^, ^23^, ^24^ We obtained the results by the Beckman Coulter Flow Cytometer and the CytExpert software (BD Biosciences).

### 2.7 Glucose uptake

We measured the glucose concentrations in cell culture supernatant by a glucose uptake assay test according to the manufacturer's protocol and previous research (GAHK20; Sigma). Results normalization was carried out according to the number of cells.

### 2.8 Gene knockdown

We used the small interfering RNA (siRNA; RiboBio, China) to knockdown specific gene. First, Lipofectamine RNAiMAX and siRNA (100 pmol) were gently mixed via pipetting and incubated at 37°C for 0.5 h. Then we changed the standard cell culture medium to transfection cocktail and incubated the cells for 6 h for siRNA gene transfection. Next, we changed the transfection cocktail to fresh cell culture medium. After 48 h, kidney cells were incubated with IL‐22 for further studies.

### 2.9 Western blot

We extracted total proteins from cell or tissue by RIPA lysis buffer (Beyotime Biotechnology, China). Then, we collected the lysates by centrifugation at 4°C at 12 000 rpm for 10 min. Subsequently, the lysates were heated with the sample buffers at 100°C for 15 min, subjected to sodium dodecyl sulfate polyacrylamide gel‐electrophoresis (SDS–PAGE), and electro transferred to polyvinylidene difluoride (PVDF) membrane (Millipore, Germany). The membranes were then blocked with 5% bovine serum albumin (BSA) for 2 h and co‐incubated with primary antibodies at 4°C overnight. After washing three times, the membranes were incubated with horseradish peroxidase–conjugated secondary antibodies (CST, USA). The protein blots were visualized by the enhanced chemiluminescence imaging system.

### 2.10 Immunofluorescence

The kidney cells were fixed by 3%–4% paraformaldehyde and permeabilized with 0.1% Triton X‐100. Then, we blocked the cells with 5% BSA for 2 h. Cells were incubated overnight with anti‐GLUT1 antibody at 4°C, washed four times, and then stained with PKH26. Subsequently, kidney cells were stained with Hoechst 33342 and mounted on slides. Data were obtained by the confocal microscopy.

### 2.11 RNA‐seq and bioinformatics

TECs samples (2 × 10^7^) were lysed and total RNA was extracted using the RNeasy mini kit, as per manufacturer’s protocols (Beyotime, Shanghai, China). Eukaryotic RNA‐seq was preformed utilizing the technology of next‐generation sequencing (NGS) on the Illumina HiSeq sequencing system, providing 50‐base read length. Alignment of sequencing reads were done utilizing TopHat v2.0.12 against the UCSC mm10 Assembly. Differential expression analysis was calculated using DESeq2 (http://www.bioconductor.org/packages/release/bioc/html/DESeq2.html) package and the statistical significance analyses were performed in R (v3.1.1; http://www.r‐project.org/). Pathway overrepresentation were performed using R package ReactomePA.

### 2.12 Statistics

Data were expressed as mean ± standard deviation (SD) and were shown using GraphPad Prism 5.0. For results comparing two or more groups, differences were calculated using the Student’s *t*‐test or the one‐way analysis of variance (ANOVA): ***p < 0.001, **p < 0.01, and *p < 0.1.

## 3 RESULTS

### 3.1 IL‐22 preserves cellular metabolism in TECs

**FIGURE 1:**
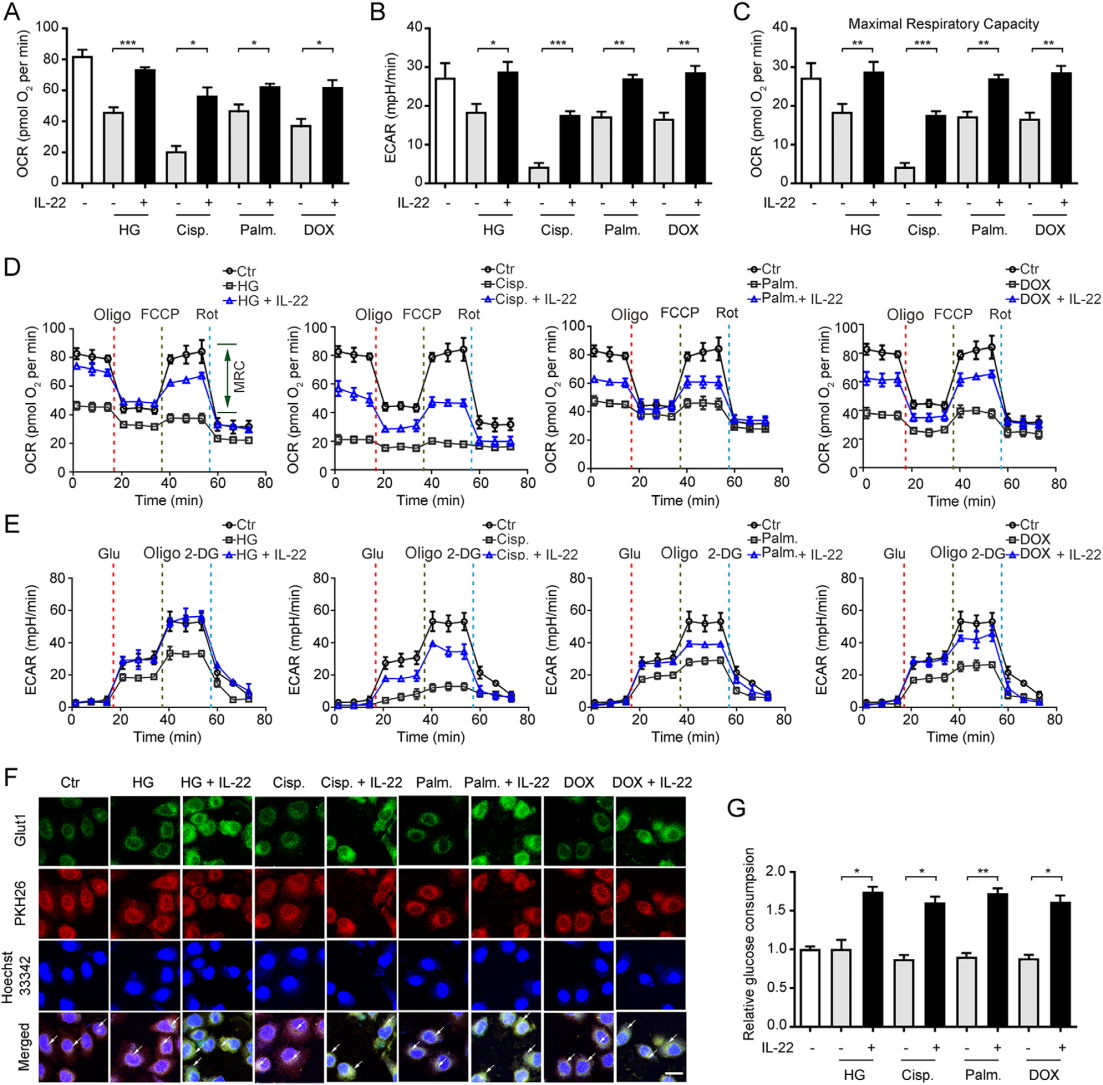
IL‐22 preserves mitochondrial fitness and glycolysis in TECs. (A and B) We use Seahorse XF96 Extracellular Flux Analyzer to test the changes in the extracellular acid rate (ECAR) and oxygen consumption rate (OCR) of TECs. OCR and ECAR in TECs incubated with 50 mM glucose, or 5 µg/mL cisplatin, or 0.2 mM palmitic acid, or 4 µM DOX in the presence or absence of IL‐22. (C) Maximal respiratory capacity (MRC) of TECs assessed by real‐time changes in OCR. (D and E) Curves in the OCR and ECAR of TECs after incubated with oligomycin, FCCP, glucose, rotenone, and 2‐DG. (F) Localization and expression of nuclear (blue), Glut1 (green), and plasma membrane (red) in TECs. (G) Glucose consumption in TECs after IL‐22 treatment for 24 h. *n* = 3; scale bars, 20 µm; Student's *t*‐test (unpaired); ***p < 0.001, **p < 0.01, *p < 0.05

Because mitochondria had been suggested to be important organelles that integrated cellular metabolism and apoptotic processes, we assessed mitochondrial function in TECs that were stimulated with kidney injury factors and treated with IL‐22.^25^ As shown in Figure 1A–D, mitochondrial basal oxygen consumption rate (OCR), maximal respiratory capacity (MRC), and respiration were blocked substantially in the TECs under stress situations. Notably, these abnormalities were largely prevented by IL‐22 treatment. Glycolysis in damaged TECs, evidenced by extracellular acidification rate (ECAR), was also markedly restrained, compared with IL‐22 plus stimuli‐challenged TECs (Figure 1B and E). We further demonstrated the compromised oxidative phosphorylation (OXPHOS) and glycolysis in damaged TECs was not the secondary effect of apoptosis via co‐treating a pan‐caspase inhibitor (Figure S1). And IL‐22‐mediated reparative effects on metabolic defects were specific for renal TECs (Figure S2). Additionally, IL‐22 promoted HK2 cells OXPHOS and glycolysis in the absence of stimuli, indicating IL‐22′s effects on metabolic pathways were not specific for damaged TECs (Figure S3). Moreover, our results also indicated that IL‐22 promoted the expression and translocation of IL‐22R1. Meanwhile, the phosphorylation of STAT3 and metabolic reprogramming effects of IL‐22 were completely disarmed by IL‐22R1 knockdown (Figure S4). These findings suggested that IL‐22 restored the metabolic state of TECs via activating IL‐22R1 directly.

Because multiple evidences had suggested that Glut1 played a principal role in glycolysis and glucose homeostasis,^26^ we, therefore, asked whether IL‐22 affected Glut1 translocation or expression. As anticipated, we demonstrated that IL‐22 not only promoted the translocated Glut1 to the cell membrane surface (PKH26) and Glut1 expression, but also enhanced glucose uptake (Figure 1F and G). Overall, these observations indicated that IL‐22 could enhance OXPHOS and glycolysis in the injured TECs.

### 3.2 IL‐22 ameliorates the accumulation of dysfunctional mitochondria in TECs via the activation of mitophagy

**FIGURE 2:**
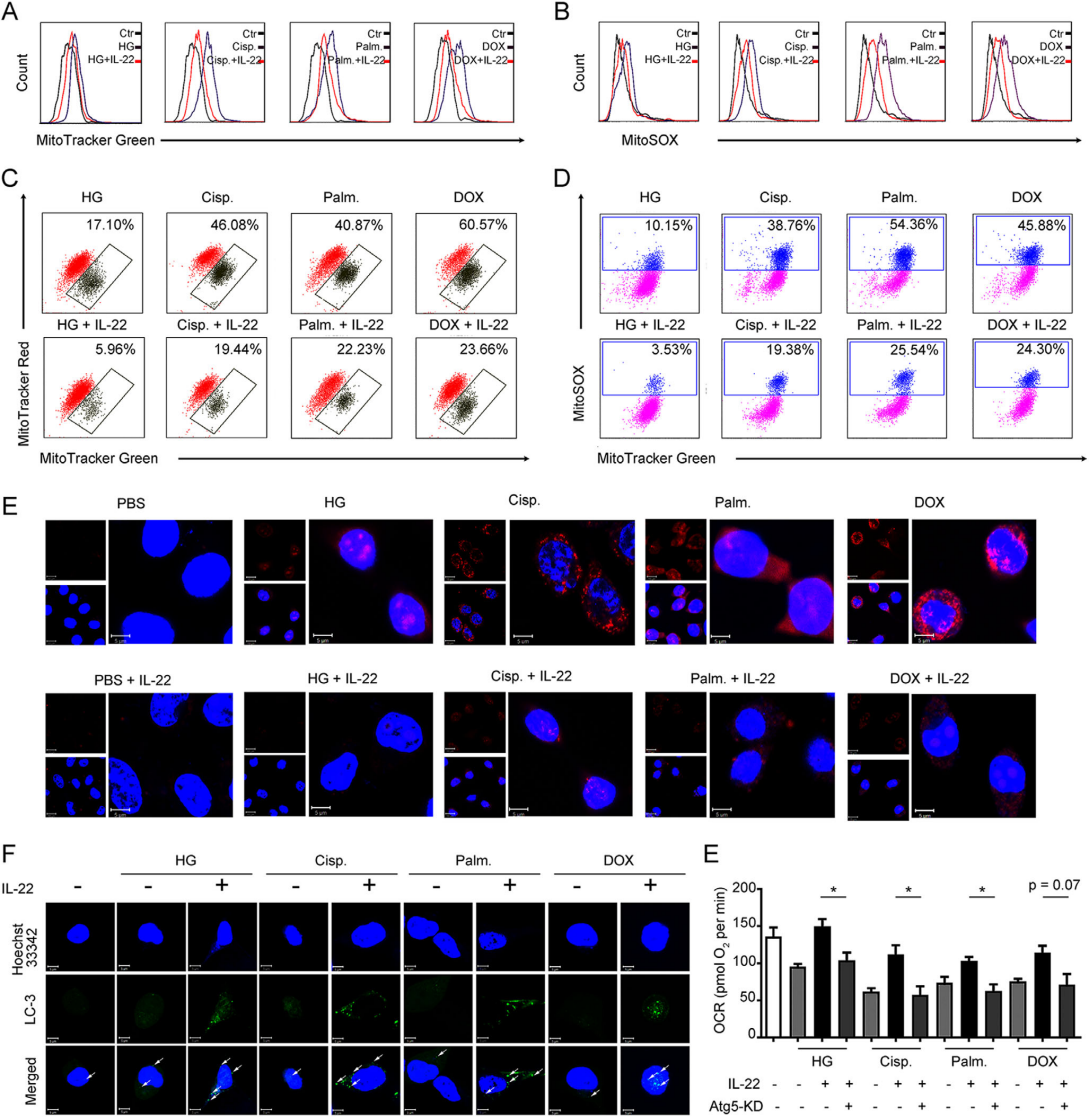
IL‐22 inhibits mitochondrial dysfunction and mitochondrial ROS accumulation via the induction of mitophagy. (A) Mitochondrial mass was stained and assessed by MitoTracker Green and flow cytometry. Mitochondrial ROS was evaluated in TECs stained with MitoSOX (B). Mitochondrial membrane potential was assessed in TECs stained with MitoTracker Green and MitoTracker Red (C); mitochondrial ROS accumulation was evaluated by MitoTracker Green and MitoSOX (D). (E) Confocal image results suggested mitochondrial ROS production labeled with MitoSOX. (F) Representative microscopic images indicated LC3‐GFP punctate formation in the presence or absence of IL‐22 TECs stimulated as (A) for 24 h. (E) OCR was assessed in the presence or absence of IL‐22 and siRNA‐ATG5 for 24 h. *n* = 3; Student's *t*‐test (unpaired); ***p < 0.001, **p < 0.01, *p < 0.05

We next investigated whether the improvement of cellular metabolism by IL‐22 in TECs was attributed to the prevention of mitochondrial dysfunction. As detected by MitoTracker Green and MitoSOX dye, we found that TECs had increased mitochondrial mass and mitochondrial ROS after exposure to stimuli (Figure 2A and B). To distinguish respiring and dysfunctional mitochondria, the TECs were then stained with MitoTracker Red to detect the mitochondrial membrane potential. Our results revealed that kidney injurious stimuli, such as cisplatin, significantly increased the mitochondria dysfunction (with higher MitoTracker Green and lower MitoTracker Red) (Figure 2C). Importantly, IL‐22 could preserve mitochondrial fitness in the injured TECs, which was showed by the reduction of mitochondrial mass, mitochondrial ROS, and mitochondrial dysfunction (Figure 2A–C). These observations corresponded to the flow cytometry data and live‐cells fluorescence images, wherein the ROS generation and accumulation could be inhibited by IL‐22 (Figure 2D and E). Of note, IL‐22‐mediated mitochondrial fitness could not be found on the nonrenal epithelial cells, suggesting its protective effects were specific for renal TECs (Figure S2). Because mitophagy played an important role in metabolic processes through restoring metabolic homeostasis and mitochondrial fitness,^27^ we, therefore, asked whether IL‐22 alleviated mitochondrial dysfunction via induction of mitophagy. Mitophagy was measured by assessing LC3‐GFP puncta generation (Figure S5). As expected, IL‐22 treatment had increased the formation of LC3 puncta in TECs after exposure to stimuli (Figure 2F). We then depleted mitophagy to assess its function on the tubule cell metabolism via siRNA‐ATG5 (Figure S6). Our data suggested that the depletion of mitophagy in TECs significantly inhibited the protective effects of IL‐22 in preserving mitochondrial fitness (Figure 2E), indicating that IL‐22 prevents mitochondrial dysfunction via the activation of mitophagy.

### 3.3 IL‐22 maintains mitochondrial integrity of TECs via activation of AMPK/AKT signaling

**FIGURE 3:**
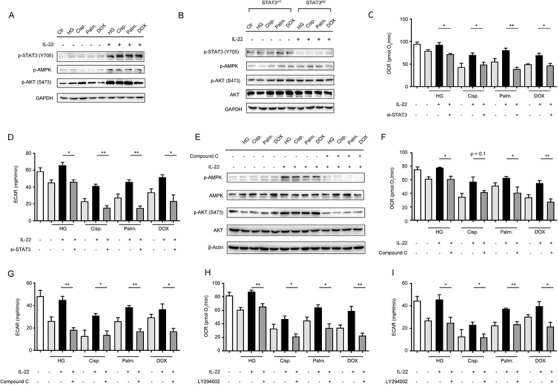
Induction of AMPK/AKT signaling by IL‐22 maintains mitochondrial fitness. TECs were stimulated 50 mM glucose, or 5 µg/mL cisplatin, or 0.2 mM palmitic acid, or 4 µM DOX in the presence or absence of IL‐22, Compound C, or LY294002 for indicated times. (A and B) AMPK/AKT signaling pathway activation in TECs or STAT3‐WT and STAT3–KD TECs was evaluated by western blot analysis. (C and D) Changes in the OCR and ECAR of TECs were analyzed. (E) AMPK/AKT signaling pathway activation in TECs was evaluated by western blot analysis. (F–I) Changes in the OCR and ECAR of TECs were measured in TECs versus Compound C and LY294002 treated TECs. Student's *t*‐test (unpaired); *n* = 3; ***p < 0.001, **p < 0.01, *p < 0.05

The AMPK signaling, as a key signaling hub linking cell survival and metabolism, controlled glucose metabolism, lipid synthesis, and mitochondrial function.^28^ According to the renal protective effect of interleukin‐22 mediated by metabolic reprogramming of TECs, we assessed whether IL‐22 regulated mitochondrial integrity via activation of AMPK signaling transduction. We found that IL‐22 increased the activity of STAT3/AMPK/AKT signaling transduction in the injured TECs, evidenced by the phosphorylation of STAT3, AMPK, and AKT (Figure 3A). Additionally, these processes were blocked in tubular cells lacking STAT3, which demonstrated that IL‐22 regulated AMPK/AKT signaling through STAT3 (Figure 3B and C; Figure S7). We further incubated TECs with compound C and LY294002 to directly inhibit AMPK/AKT signaling transductions, and then evaluate their mitochondrial functions and metabolism states. The results revealed that co‐treatment with these signaling inhibitors significantly prevented the improved OXPHOS and glycolytic flux induced by IL‐22 treatment (Figure 3D–I), which suggested that IL‐22 promoted TECs metabolism via activating AMPK/AKT signaling pathway.

### 3.4 IL‐22 preserves cellular metabolism and mitochondrial integrity of TECs via induction of PFKFB3

**FIGURE 4:**
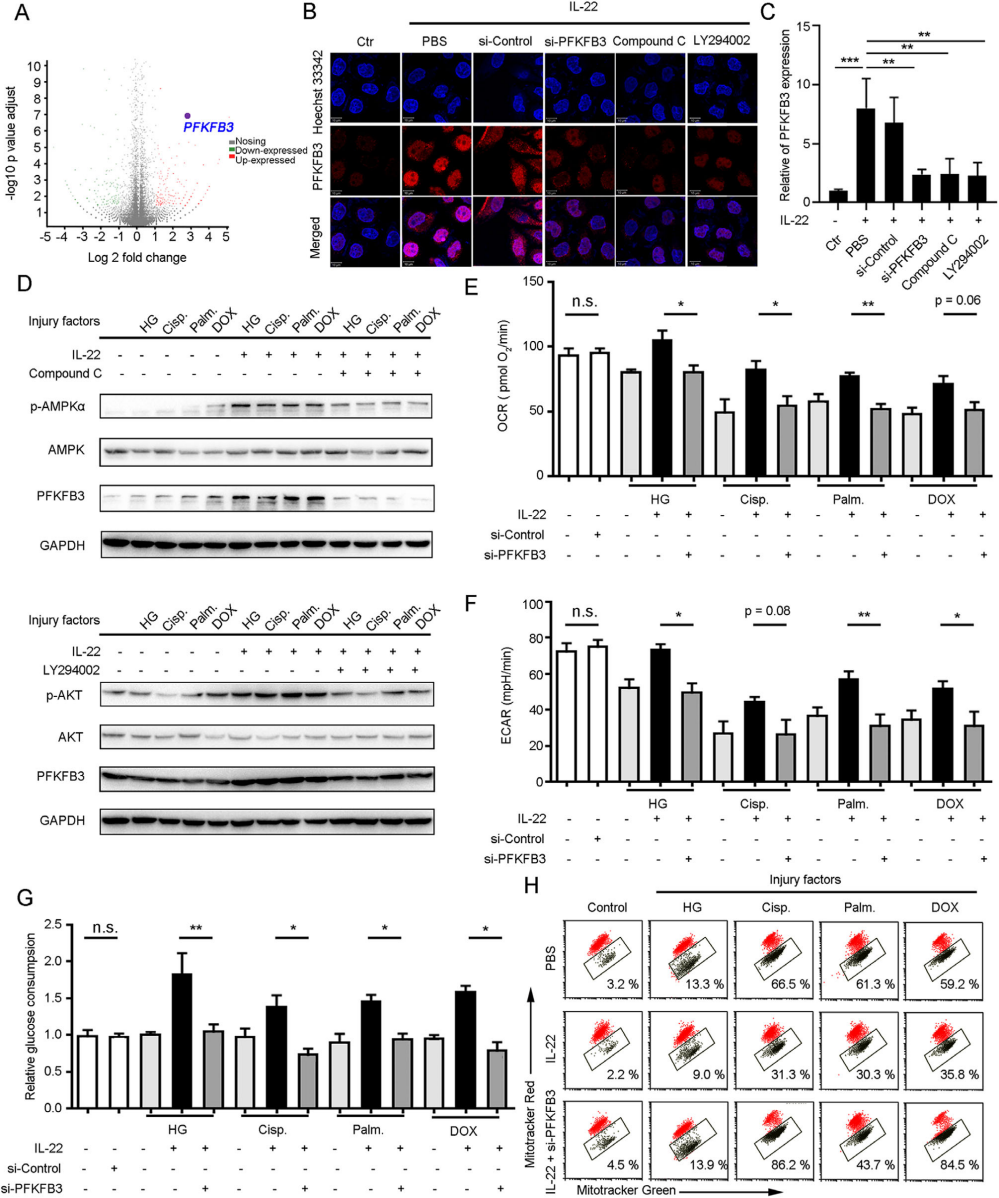
IL‐22 maintains mitochondrial integrity and cellular metabolism of TECs via induction of PFKFB3. (A) Volcano plot for genes expression in TECs stimulated with glucose, or cisplatin, or palmitic acid, or CCl_4_ in the presence or absence of IL‐22 for 24 h. (B) Confocal microscopic data demonstrated PFKFB3 overexpression in TECs after IL‐22 treatment, which required AMPK/AKT signaling activation. (C) Real‐time PCR results indicating that IL‐22 induced PFKFB3 overexpression in TECs, which could be inhibited by AMPK/AKT signaling blocked. (D) The AMPK/AKT/ PFKFB3 signaling pathway activation in TECs after treated with Compound C and LY294002. (E and F) ECAR and OCR in TECs at the presence or absence of IL‐22, or si‐PFKFB3 for 24 h. (G) Glucose consumption in TECs after IL‐22 or si‐PFKFB3 treatment for 24 h. (H) Mitochondrial dysfunction was evaluated in TECs stained with MitoTracker Red and MitoTracker Green. Student's *t*‐test (unpaired); *n* = 3; ***p < 0.001, **p < 0.01, *p < 0.05

Moreover, to explore how TECs’ mitochondrial fitness and metabolism were improved, we used RNA sequencing analysis (RNA‐seq) to assess gene expression following stimuli injury and IL‐22 treatment. We demonstrated that multiple genes were significantly changed in TECs after IL‐22 incubation (Figure 4A). Using the available databases, the gene set enrichment analysis of those up‐regulated genes was performed, which suggested a remarkable enrichment of genes in the AMPK signaling pathway and MYC targets signaling pathway (Figure S8). Notably, PFKFB3 was significantly induced by IL‐22 from among the genes during kidney injury factors stimulation (Figure 4A). These observations were also indicated by confocal images, Q‐PCR, and western blot. The high expression of PFKFB3 was AMPK/AKT signaling dependent (Figure 4B and C; Figure S9).

Previous studies had suggested that PFKFB3 could be regulated by numerous signaling transductions to accelerate epithelial regeneration and repair following injury.^29^, ^30^ Thus, we speculated that IL‐22 induced PFKFB3 to preserve the cellular metabolism of TECs and maintain their mitochondrial fitness. We observed that the inhibition of AMPK/AKT signaling reduced PFKFB3 expression (Figure 4D). So, we silenced PFKFB3 to further study its functions in TECs after IL‐22 incubation. Our findings indicated that silenced PFKFB3 alleviated the metabolic reprogramming effects of IL‐22 on restoring ECAR and OCR (Figure 4E and F), and prevented glucose uptake as well as dysfunctional mitochondria (Figure 4G and H). Moreover, we further demonstrated that the metabolic reprogramming effects of IL‐22 were completely disarmed by STAT3‐knockdown while the overexpression of PFKFB3 could be able to rescue these phenotypes, suggesting IL‐22 restored the metabolic state of damaged kidney epithelial cells via targeting STAT3‐AMPK/AKT‐PFKFB3 signaling directly (Figure S10). Taken together, these findings indicated that the activation of PFKFB3 played an important role in mitochondrial fitness and dysfunctional mitochondria elimination after kidney injury.

### 3.5 IL‐22 alleviates kidney injury in cisplatin‐induced AKI via suppression of renal ROS accumulation and mitochondrial dysfunction

**FIGURE 5:**
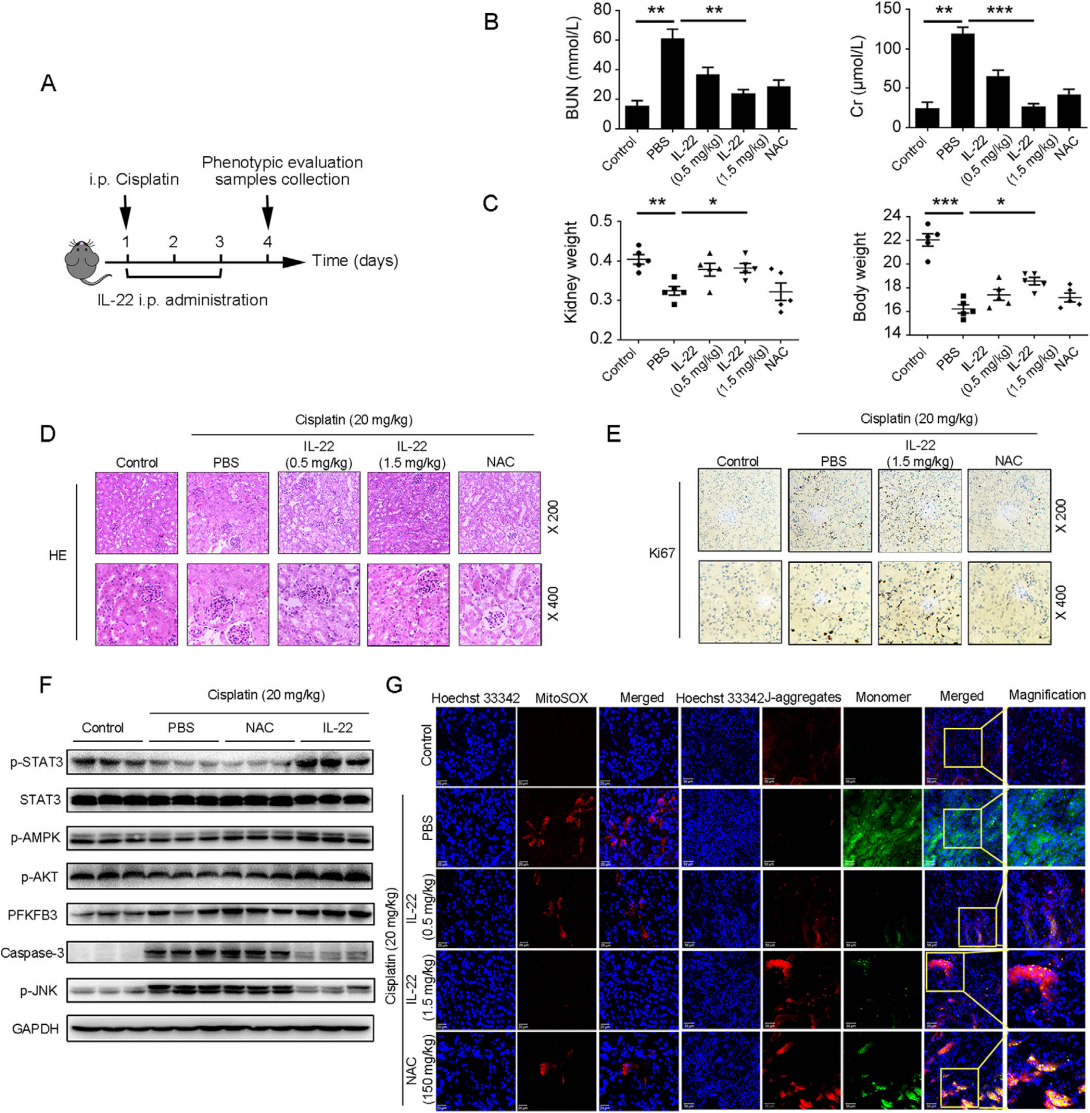
IL‐22 alleviates kidney damage in cisplatin‐induced AKI via inhibition renal ROS accumulation and dysfunctional mitochondria. (A) Schematic diagram of the experimental protocols to evaluate the protective effects of IL‐22 in cisplatin (20 mg/kg) induced kidney damage. PBS as a vehicle control group and *N*‐acetyl‐l‐cysteine (NAC, 150 mg/kg) as a positive control group. (B and C) Serum BUN levels, serum Cr levels, kidney weights, and body weights were assessed. Representative HE (D), Ki‐67 staining (E), MitoSOX (G), and JC‐1 (G) images of the kidney sections were presented. (F) Comparison of STAT3/AMPK/AKT/PFKFB3 activation in kidney extracts from the IL‐22‐treated mice was evaluated by western blot. Student's *t*‐test (unpaired); *n* = 3; ***p < 0.001, **p < 0.01, *p < 0.05

To test our hypothesis that IL‐22 regulated renal metabolic profiles to suppress kidney tubule injury in vivo, we initially evaluated renal functions in IL‐22‐treated mice that had been administered with cisplatin (Figure 5A). Four days after cisplatin injection, IL‐22 not only substantially reduced tubular cellular damage and hemorrhage; their renal dysfunction also had been significantly attenuated, which were assessed by the serum levels of blood urea nitrogen (BUN) and creatinine (Cr) (Figure 5B–D). We further suggested IL‐22 prevented kidney injury via enhancing kidney regeneration and activating AMPK/AKT signaling and PFKFB3 (Figure 5E and F; Figure S11). Next, we employed MitoSOX (mitochondria‐specific ROS dye) and JC‐1 (mitochondrial membrane potential‐dependent dye) to assess the mitochondrial dysfunction involved in cellular metabolism and apoptosis. In the kidney sections of mice that had been treated with cisplatin, there were comparable accumulations of ROS. Of note, at four days after IL‐22 treatment, the levels of ROS were markedly reduced in the injured kidneys in comparison to ROS from mice after PBS treatment (Figure 5G). Taken together, these observations offered evidence that IL‐22 played a protective role in cisplatin‐induced AKI via controlling mitochondrial fitness.

### 3.6 IL‐22 ameliorates diabetes‐induced renal injury via inhibition of mitochondrial dysfunction through the activation of AMPK/AKT signaling and PFKFB3

**FIGURE 6:**
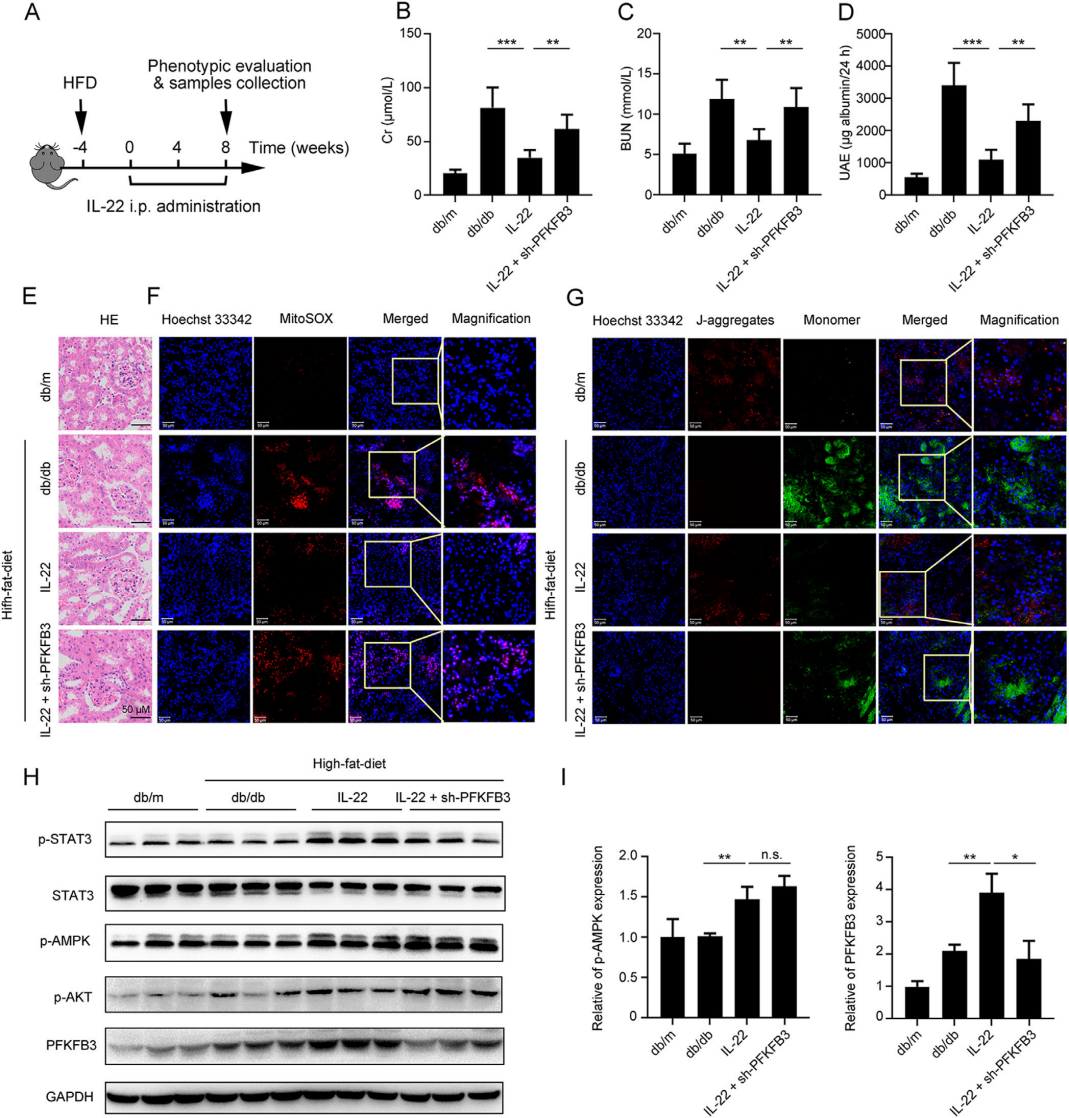
IL‐22 alleviates diabetes‐induced renal damage through AMPK/AKT signaling and inhibition of dysfunctional mitochondria via activation of PFKFB3. (A) Schematic diagram of the experimental protocols to evaluate the protective effects of IL‐22 in diabetes‐induced renal damage. Db/m as a vehicle control group. (B–D) Serum BUN levels, serum Cr levels, and UAE were evaluated. Representative HE (E), MitoSOX (F), and JC‐1 (G) images of the kidney sections were presented. (H and I) Comparison of STAT3/AMPK/AKT/PFKFB3 activation in kidney extracts from IL‐22‐treated mice was evaluated by western blot. Student's *t*‐test (unpaired); *n* = 3; ***p < 0.001, **p < 0.01, *p < 0.05

We and others had demonstrated that IL‐22 could exert protective potency in diabetic nephropathy (DN), which was the main cause of ESTD.^31^, ^32^, ^33^ To further study the underlying mechanisms, we fed db/db mice with high‐fat diets (HFD) and then treated with IL‐22 (2.5 mg/kg, ip) for 8 weeks (Figure 6A). When mice were fed with HFD only, we found kidney tubular fibrosis and dilatation, in addition to atrophy. In contrast, when mice were treated with IL‐22, renal fibrosis and histopathology were significantly improved, as well as kidney functions. Enhanced kidney functions were also demonstrated by decreased serum Cr, BUN levels, and total urinary albumin/24 h (UAE) levels (Figure 6B–E). To study whether these activities were associated with mitochondria, we assessed the mitochondrial function involved in cellular metabolism and apoptosis. The results indicated that the increased mitochondrial ROS levels and accumulated dysfunctional mitochondria were significantly alleviated by IL‐22 treatment (Figure 6F and G). To further study whether the protective functions of IL‐22 in the kidney were mediated through PFKFB3, we injected mice with PFKFB3 shRNA adenovirus to knockdown PFKFB3. Our data indicated that the beneficial effects of IL‐22 on HFD‐induced renal injury, necrosis, steatosis, mitochondrial dysfunction, and ROS accumulation and the activation of related signaling pathways were significantly blocked by PFKFB3 knockdown (Figure 6B–G). The increased expression of AMPK/AKT signaling was also observed after treatment with IL‐22 (Figure 6H). Along with the alleviation of kidney dysfunction, the expressions of PFKFB3 and important enzymes in cellular metabolism were also improved in the kidneys of IL‐22‐treated mice (Figure 6H and I). Additionally, histological analysis on major organs indicated no morphological injuries after IL‐22 injection, suggesting IL‐22 could be a safe antidote without significant side‐effects or toxic reactions for kidney injury disorders (Figure 12S). Overall, our data suggested that IL‐22 alleviated kidney damage via activation of AMPK/AKT signaling transduction, inhibition of ROS accumulation, and mitochondrial function regulation through PFKFB3.

## 4 DISCUSSION

IL‐22 is secreted by immune cells, such as NK cells, neutrophils, innate lymphoid cells (ILCs), and T cells, but does not directly affect these cells. It predominantly regulates the functions of epithelial cells owing to the restricted IL‐22R1 expression on epithelial cells including TECs.^15^, ^34^ Previously studies have suggested that IL‐22 can protect against renal epithelial injury and accelerate tubular regeneration through inhibition of NLRP3 inflammasome and activating STAT3 and AKT signaling.^16^, ^31^ However, the underlying mechanisms of this protection are not yet to be elucidated. To further determine the molecular basis of IL‐22′s function in kidney injury, we first investigate whether IL‐22 can alleviate kidney cell dysfunction and subsequent apoptosis through regulating their metabolic states. Moreover, we study two models of kidney injury diseases (AKI and DN) with IL‐22 administration and compare the observations with those of control groups. We indicate that IL‐22 corrects metabolic reprogramming to maintain mitochondrial integrity, decreases mitochondrial ROS accumulation in injured TECs, and alleviates progressive kidney damage and necrosis. Moreover, our findings suggest that IL‐22 can activate AMPK/AKT associated signaling pathways in TECs and kidneys, important mediators of epithelial wound healing and cell survival, to ameliorate mitochondrial dysfunction and the deteriorating metabolic profiles and opening up a novel field for IL‐22 mediated renal protective mechanism. Overall, our results indicate that the renal protective effects of IL‐22 are mediated via metabolic reprogramming processes.

The critical role of OXPHOS and glycolysis in kidney protection have been revealed in previous studies, where balancing fuel utilization by inhibitory S‐nitrosylation of pyruvate kinase M2 (PKM2) protects against kidney injury.^19^ Our results, suggesting the promotion of OXPHOS and glycolysis by IL‐22 via increasing metabolic regulators expression and glucose uptake, demonstrate that IL‐22 reverses the deteriorating metabolic states associated with the kidney injury. These observations are consistent with the renal protective functions of recombinant irisin and also in line with findings from hepatocytes, where both OXPHOS and glycolysis are needed for cellular functions, but if these are inhibited, then the primary tubule cells and hepatocytes become dysfunctional.^35^, ^36^

Mitochondrial dysfunction has emerged as the key molecular basis that integrates metabolic profiles and cell death. Herein, we illustrate that upon injury factors stimulation, TECs treatment with IL‐22 had ameliorated accumulation of mitochondrial ROS and dysfunctional mitochondria as compared with the decreased mitochondrial integrity and function in control groups. Importantly, AMPK signaling pathways, critical energy sensors, modulate metabolic processes to maintain cellular homeostasis and prevent cell or tissue damage.^37^, ^38^, ^39^ In the current study, our observations indicating that IL‐22 preserves mitochondrial integrity and function via activating the AMPK/AKT signaling demonstrate that IL‐22 prevents mitochondrial dysfunction in a direct method, which is vital to maintaining TECs respiratory capacity. Furthermore, we demonstrate that IL‐22 signaling through STAT3 has direct effects on preserving mitochondrial fitness, as those have been investigated previously that induced STAT3 phosphorylation is critical for preserving mitochondrial integrity.^40^, ^41^

**FIGURE 7:**
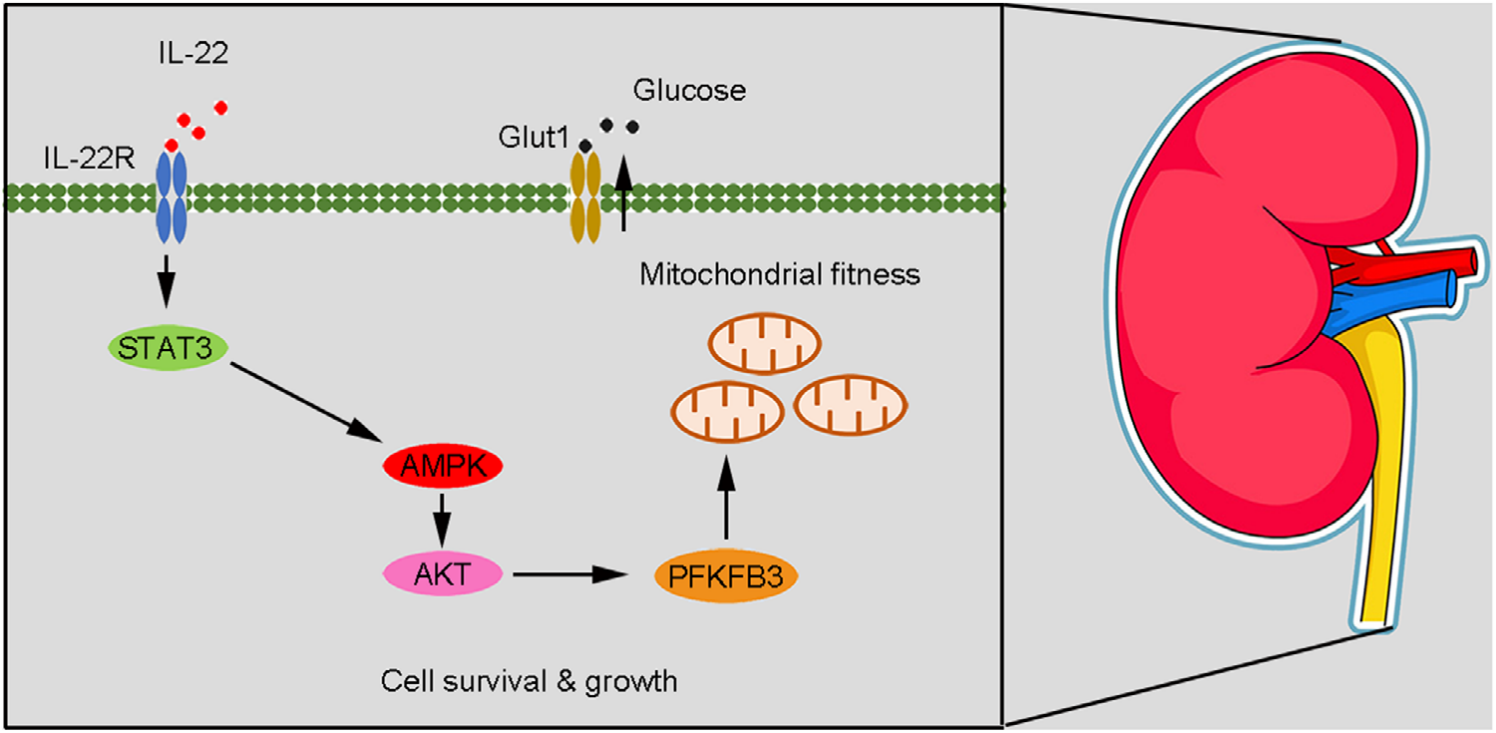
Our findings illustrate an important role of IL‐22 in controlling kidney cell metabolism to treat kidney damage through activating the STAT3‐AMPK/AKT‐PFKFB3 axis. These results suggest that the therapeutic opportunities of IL‐22 and targeting associated metabolic signaling could be directly favorable for numerous kidney damage treatment or prevention

Multiple literatures have previously indicated PFKFB3 plays the part of a central modulator in cell metabolism.^29^, ^30^, ^42^ Herein, we first demonstrate that IL‐22 promotes PFKFB3 activation through AMPK/AKT signaling in TECs, suggesting IL‐22 regulates metabolic states via involving the control of PFKFB3. Moreover, previous studies further indicate that PFKFB3 is important to control the exaggerated OXPHOS and glycolysis in lots of cells or tissues.^42^, ^43^ Consistent with these studies, our observations also indicate promotion of OXPHOS and glycolysis in the IL‐22‐treated TECs via the activation of PFKFB3. Among numerous downstream modulators of IL‐22 that have been investigated, our data illustrate that PFKFB3 is significantly upregulated after IL‐22 treatment during TECs damage and that the STAT3–AMPK/AKT‐PFKFB3 axis is vital for the preservation of mitochondrial integrity during kidney injury (Figure 7). They remain to be investigated if IL‐22 also regulates other metabolic regulators and processes related to kidney injury through activating the STAT3–AMPK/AKT‐PFKFB3 signaling pathway.

## 5 CONCLUSIONS

In conclusion, we demonstrated the therapeutic potential and underlying mechanisms of IL‐22 in kidney damage. IL‐22 regulated renal cell metabolism via metabolic reprogramming to preserve mitochondrial integrity. Inhibition of their regulators (i.e., AMPK, AKT, and PFKFB3) could lead to abnormal metabolic profiles and loss of mitochondrial fitness, as indicated in TECs with spontaneous IL‐22 treatment, which had increased dysfunctional mitochondria and mitochondrial ROS accumulation. Most importantly, the kidney damage by mitochondrial dysfunction and ROS accumulation in mice models was substantially alleviated by IL‐22 treatment through the activation of STAT3‐AMPK‐AKT‐PFKFB3 signaling. Thus, our study indicated that targeting associated metabolic signaling could be directly favorable for numerous kidney damage diseases and IL‐22 is a potential therapeutic agent for preventing and treating these diseases.

## AUTHOR CONTRIBUTIONS

Wei Chen, Zhen Zhao, and Dianwen Ju designed research, analyzed data, and wrote the paper; Wei Chen, Yilan Shen, Jiajun Fan, Xian Zeng, Xuyao Zhang, Jingyun Luan, Yichen Wang, Jinghui Zhang, and Si Fang performed research.

## CONFLICT OF INTEREST

The authors declare no conflict of interest.

## COMPLIANCE WITH ETHICS GUIDELINES

All national guidelines and institutional for the use and care of laboratory animals were followed.

## Supporting information



Supporting Information
