# Supplementary material for: IL‐22‐mediated renal metabolic reprogramming via PFKFB3 to treat kidney injury
Source: Clin Transl Med. 2021 Feb 23;11(2):e324. doi: 10.1002/ctm2.324 (PMC7901723; doi:10.1002/ctm2.324)
Supplement: Supplementary file 1 — Supporting Information [file CTM2-11-e324-s001.pdf]

IL-22 mediated renal metabolic reprogramming via PFKFB3 to treat kidney injury

Wei Chen[1,2,6], Yilan Shen[1,3,5,6], Jiajun Fan[1], Xian Zeng[1], Xuyao Zhang[1], Jingyun Luan[1], Yichen Wang[1], Jinghui Zhang[1], Si Fang[4], Xiaobin Mei[3], Zhen Zhao[1*], Dianwen Ju[1*]

[1]School of Pharmacy and Minhang Hospital, Shanghai Engineering Research Center of Immunotherapeutics, Fudan University, Shanghai, P. R. China

[2]Department of Ophthalmology, Stanford University School of Medicine, Palo Alto CA 94304, USA

[3]Changhai Hospital, Second Military Medical University, Shanghai, 200433, P. R. China

[4]Tongcheng Hospital of Traditional Chinese Medicine, Anhui, 231400, P. R. China

[5]Department of Nephrology, Shanghai Jiao Tong University Affiliated Sixth People's Hospital, Shanghai 200233, China

[6]These authors contributed equally to this work

*Correspondence to:

Prof. Zhen Zhao, Email: fdmh_zz@fudan.edu.cn, Minhang Hospital, Fudan University, 170 Xinsong Road, Shanghai 201199, China

Prof. Dianwen Ju, Email: dianwenju@fudan.edu.cn, Department of Biological Medicines & Shanghai Engineering Research Center of Immunotherapeutics, Fudan University School of Pharmacy, Shanghai, 201203, P. R. China

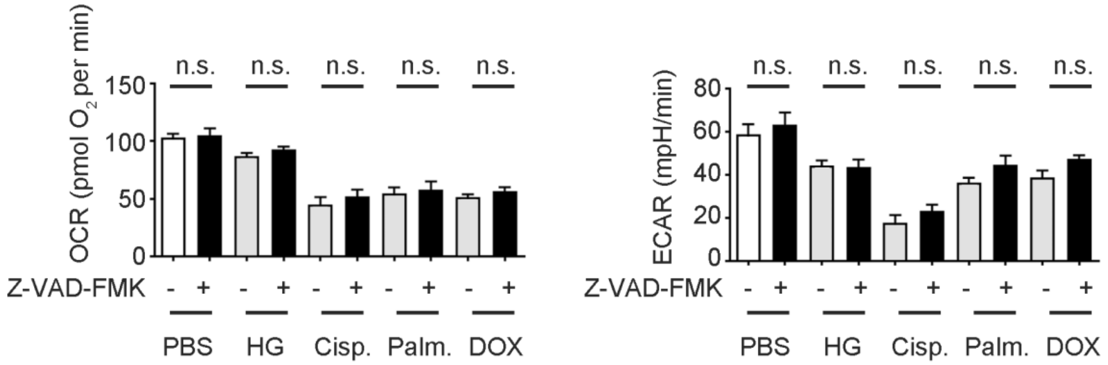

Figure S1. Seahorse XF96 Extracellular Flux Analyzer was used to test the changes in the extracellular acid rate (ECAR) and oxygen consumption rate (OCR) of TECs. OCR and ECAR in TECs incubated with 50 mM glucose, or 5 μg/mL cisplatin, or 0.2 mM palmitic acid, or 4 μM DOX in the presence or absence of Z-VAD-FMK.

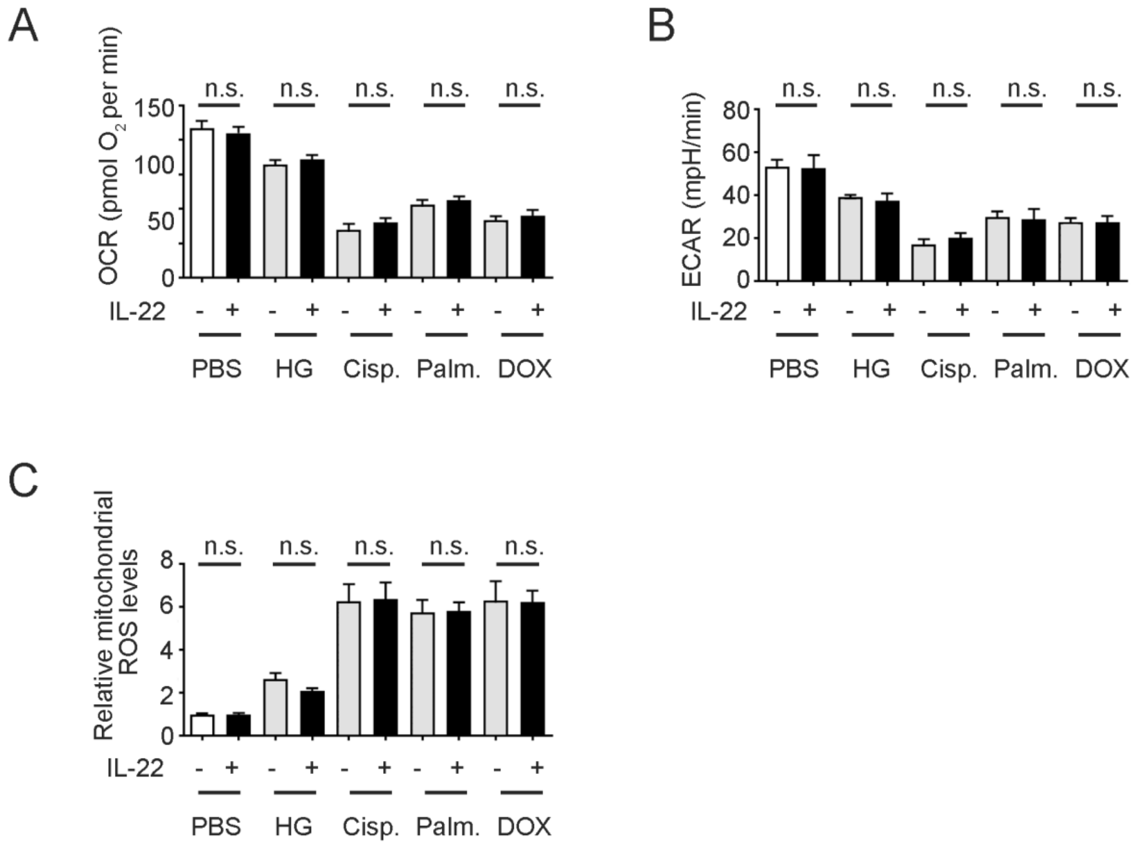

Figure S2. (**A** and **B**) Seahorse XF96 Extracellular Flux Analyzer was used to test the changes in the ECAR and OCR of a non-renal epithelial cell type (renal mesangial cell, SV40 MES 13). OCR and ECAR in non-renal epithelial cells incubated with 50 mM glucose, or 5 μg/mL cisplatin, or 0.2 mM palmitic acid, or 4 μM DOX in the presence or absence of IL-22. (**C**) Mitochondrial ROS level SV40 MES 13 cells was measured by MitoSOX.

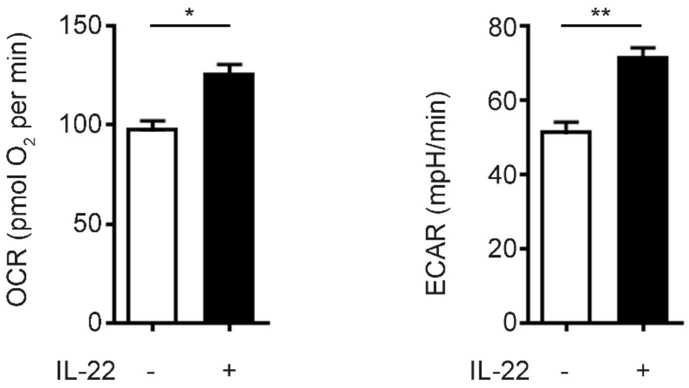

Figure S3. (**A** and **B**) OCR and ECAR in renal tubule epithelial cells treated with IL-22 for 24 h (n = 3). **P < 0.01, *P < 0.1.

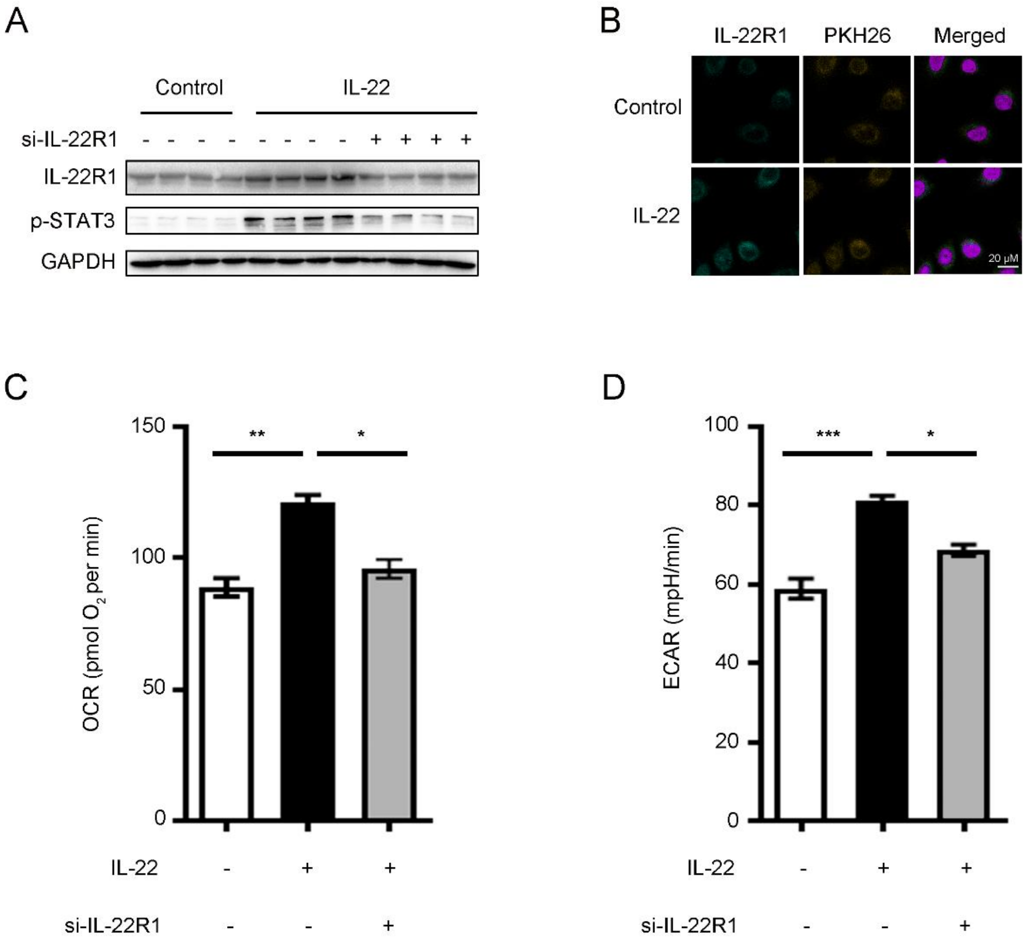

Figure S4. (**A**) TECs were treated IL-22 and siRNA of IL-22R1 (si-IL22R1). IL-22R1/STAT3

signaling pathway activation in IL22R1–KD TECs was evaluated by western blot analysis. (**B**) Localization and expression of IL22R1 in TECs. (**C** and **D**) OCR and ECAR in renal TECs treated with IL-22 and si-IL22R1 for 24 h ($n = 3$). ***$P < 0.001$, **$P < 0.01$, *$P < 0.1$.

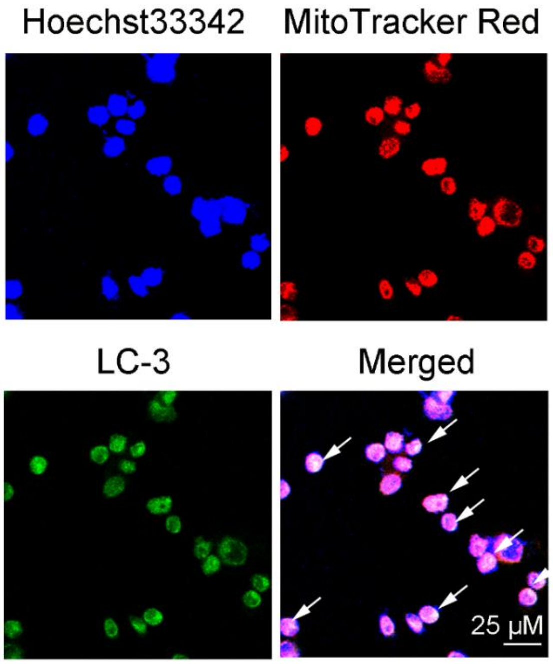

Fig. S5. Colocalization of mitochondria and LC3 punctate in TECs was measured using confocal.

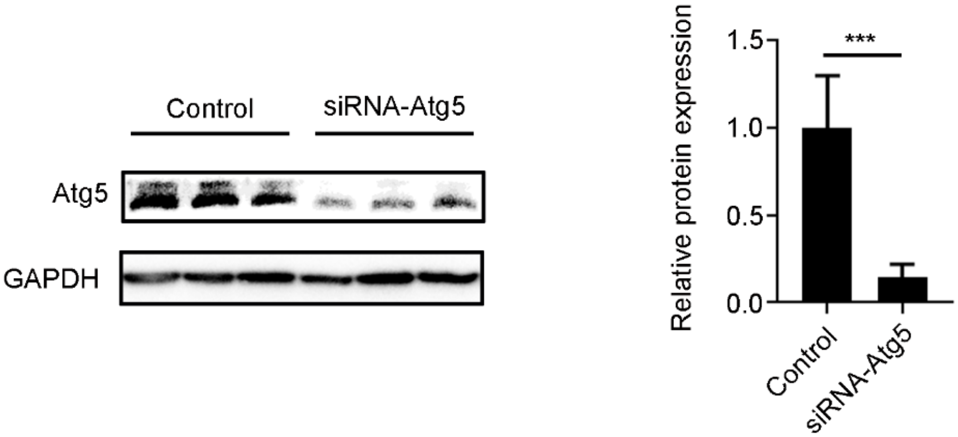

Figure S6. TECs were treated with PBS or siRNA-Atg5 for 48 h. Atg5 expression in TECs was evaluated by western blot analysis. Densitometric values were quantified and normalized to control group (n = 3; mean ± SD; ***P < 0.001). The values of control group were set to 1.

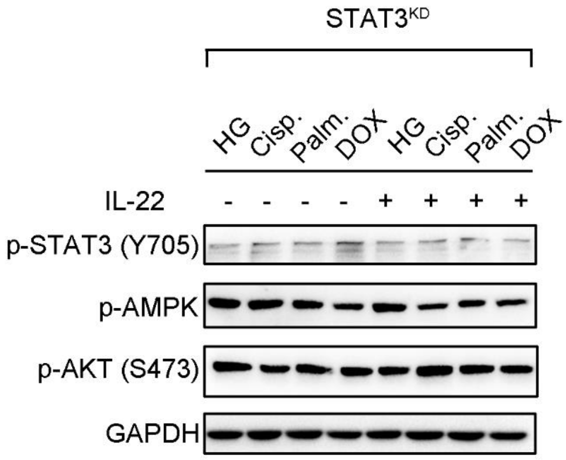

Figure S7. TECs were stimulated 50 mM glucose, or 5 μg/mL cisplatin, or 0.2 mM palmitic acid, or 4 μM DOX in the presence or absence of IL-22. AMPK/AKT signaling pathway activation in STAT3–KD TECs was evaluated by western blot analysis.

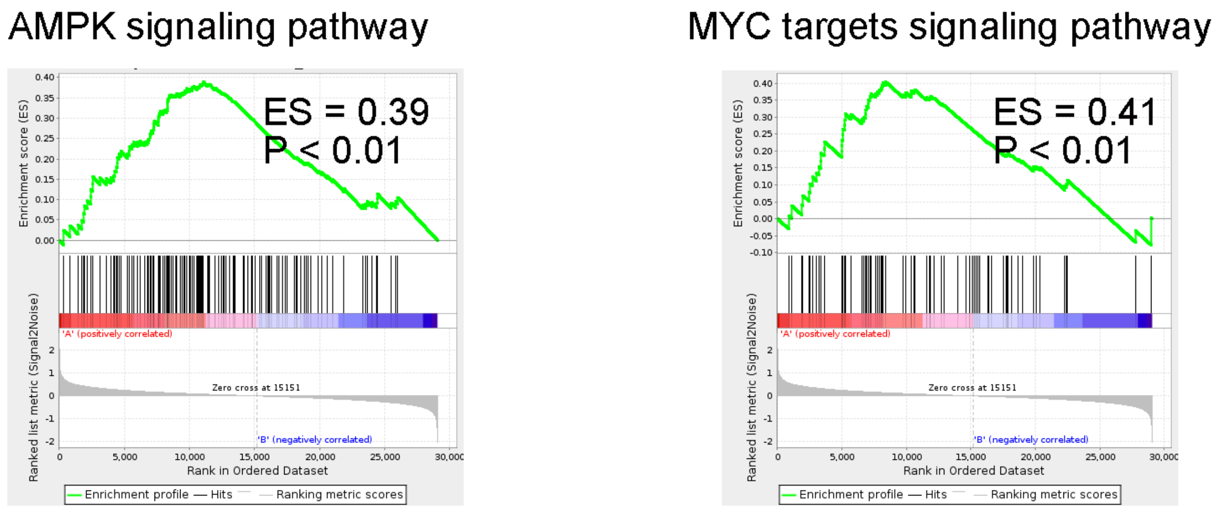

Figure S8. KEGG of AMPK signaling pathway and MYC targets signaling pathway in IL-22-protected and -nonprotected TECs.

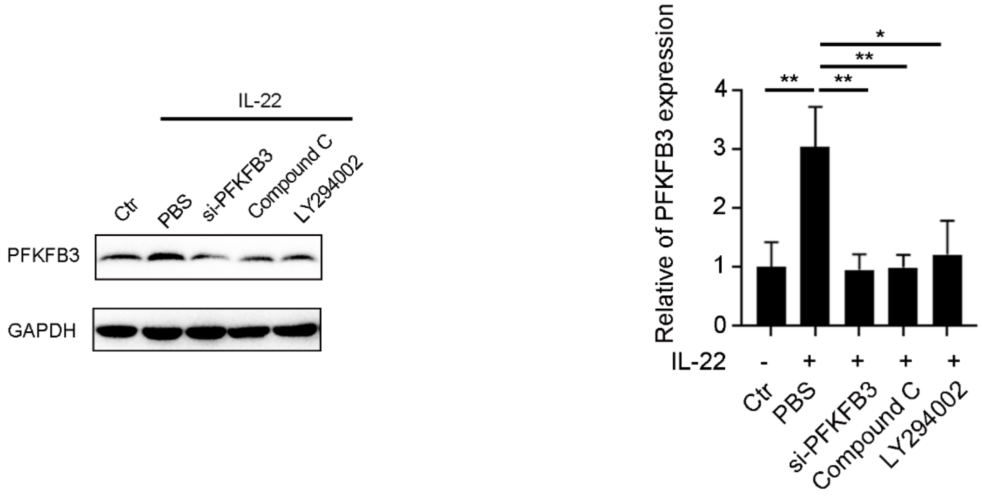

Figure S9. Kidney cells were treated with PBS or siRNA-PFKFB3, or Compound C, or LY294002 or IL-22. PFKFB3 expression was evaluated by western blot analysis. Densitometric values were quantified and normalized to control group (n = 3; mean ± SD; $**P < 0.01$, $*P < 0.1$). The values

of control group were set to 1.

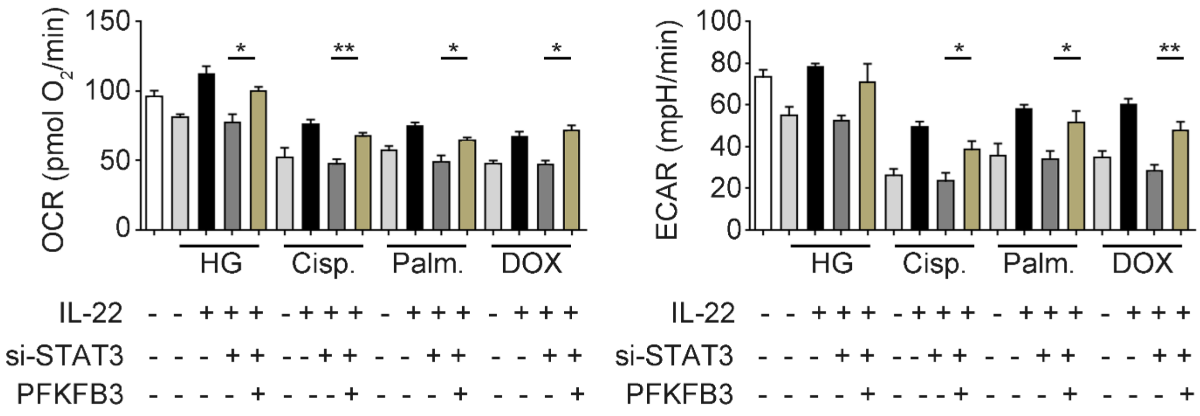

Figure S10. Basal OCR and ECAR in TECs at the absence or presence of IL-22, or si-STAT3 or PFKFB3 for 24 h (n = 3; mean ± SD; **P < 0.01, *P < 0.1).

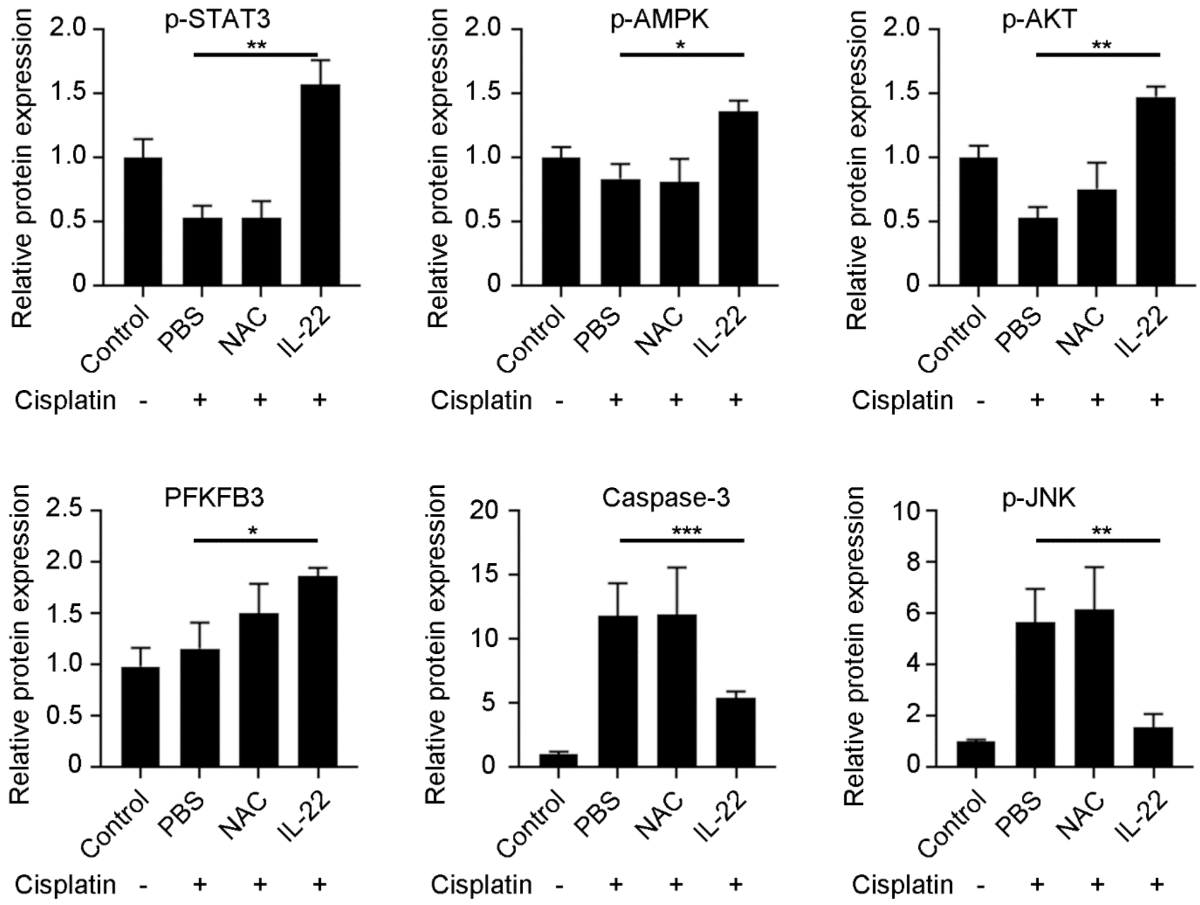

Figure S11. Densitometric values were quantified and normalized to control group (n = 3; mean ± SD; ***$P < 0.001$, **$P < 0.01$, *$P < 0.1$). The values of control group were set to 1.

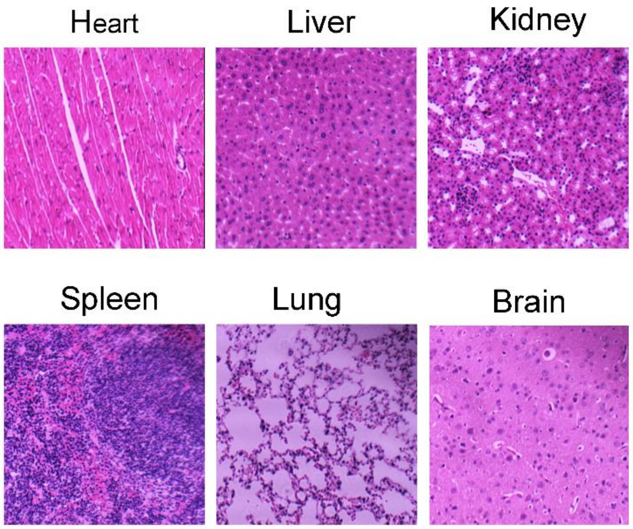

Figure S12. Histological analysis of major organs in mice after injection with IL-22 by HE-staining.
